# Defective Neutrophil Transendothelial Migration and Lateral Motility in ARPC1B Deficiency Under Flow Conditions

**DOI:** 10.3389/fimmu.2021.678030

**Published:** 2021-05-31

**Authors:** Lanette Kempers, Evelien G. G. Sprenkeler, Abraham C. I. van Steen, Jaap D. van Buul, Taco W. Kuijpers

**Affiliations:** ^1^ Molecular Cell Biology Laboratory, Department of Molecular and Cellular Haemostasis, Sanquin Research, Amsterdam University Medical Center (AUMC), Amsterdam, Netherlands; ^2^ Department of Blood Cell Research, Sanquin Research, AUMC, University of Amsterdam, Amsterdam, Netherlands; ^3^ Department of Pediatric Immunology, Rheumatology and Infectious Diseases, Emma Children’s Hospital, AUMC, University of Amsterdam, Amsterdam, Netherlands; ^4^ Leeuwenhoek Centre for Advanced Microscopy, Section Molecular Cytology, Swammerdam Institute for Life Sciences, University of Amsterdam, Amsterdam, Netherlands

**Keywords:** primary immunodeficiency, ARPC1B deficiency, ARP2/3 complex, neutrophil, neutrophil transmigration, inborn error of immunity, vessel-on-a-chip

## Abstract

The actin-related protein (ARP) 2/3 complex, essential for organizing and nucleating branched actin filaments, is required for several cellular immune processes, including cell migration and granule exocytosis. Recently, genetic defects in ARPC1B, a subunit of this complex, were reported. Mutations in *ARPC1B* result in defective ARP2/3-dependent actin filament branching, leading to a combined immunodeficiency with severe inflammation. *In vitro*, neutrophils of these patients showed defects in actin polymerization and chemotaxis, whereas adhesion was not altered under static conditions. Here we show that under physiological flow conditions human ARPC1B-deficient neutrophils were able to transmigrate through TNF-α-pre-activated endothelial cells with a decreased efficiency and, once transmigrated, showed definite impairment in subendothelial crawling. Furthermore, severe locomotion and migration defects were observed in a 3D collagen matrix and a perfusable vessel-on-a-chip model. These data illustrate that neutrophils employ ARP2/3-independent steps of adhesion strengthening for transmigration but rely on ARP2/3-dependent modes of migration in a more complex multidimensional environment.

## Introduction

Neutrophils are the most abundant type of leukocytes in the human circulation and important effector cells in the innate immune system. They are the first cells recruited to sites of infection or inflammation, where they extravasate through the blood vessel into the tissue. This process, also known as transendothelial migration (TEM), can be divided into several steps, namely tethering, selectin-mediated rolling and slow rolling, selectin-mediated and chemokine-mediated integrin activation resulting in arrest, adhesion strengthening, spreading, intravascular crawling, and transmigration (either paracellular or transcellular) (1). Once neutrophils cross the endothelial cell layer they encounter a second barrier, the vascular basement membrane (BM). This BM provides structural support for endothelial cells and is composed of a network of multiple extracellular matrix (ECM) proteins, including laminins and collagen type IV (2). After crossing these layers, neutrophils continue to migrate and enter the tissue to reach and fight infection.

By studying rare primary immunodeficiencies (PIDs), essential proteins in the different steps of TEM have been identified. Well-known PIDs resulting in defective TEM are leukocyte adhesion deficiencies (LADs), where patients have mutations in genes involved in leukocyte integrin signaling (LAD-I and LAD-III), resulting in an adhesion defect. In LAD-II, defective fucosylation of selectin ligands results in the inability of neutrophils to bind to endothelial selectins (E- and P-selectins), resulting in a rolling defect (3).

In 2017, a novel PID involving infections, bleeding episodes, allergy and auto-inflammation was identified caused by mutations in the *ARPC1B* gene (4). ARPC1B is one of 7 subunits of the actin-related protein (ARP) 2/3 complex, which is required for the formation of branched actin networks as it nucleates a daughter filament to the side of a pre-existing actin filament (5). These branched actin filaments are of vital importance for the formation of lamellipodia at the leading edge of migrating cells. Analysis of patient-derived neutrophils showed a defect in actin polymerization, resulting in a severe chemotaxis defect through 3-µm pore-size filters (4). Here we investigated ARPC1B-deficient neutrophil migration in more depth by using TEM flow and 3D vessel-on-a-chip models allowing us to monitor the full process of neutrophil migration from the vessel lumen into the tissue environment.

## Materials and Methods

### Isolation of Human Primary Cells From Patient and Controls

Heparinized venous blood was drawn from healthy controls and an ARPC1B-deficient patient after informed consent had been obtained. A detailed description of the patient’s history has been reported previously (4). Neutrophils were isolated as previously described (6). Subsequently, neutrophils (5x10^6^/mL) were fluorescently labelled with Vybrant™ DiD Cell-Labeling Solution (dilution 1:1,000; Invitrogen, Carlsbad, CA, USA) or calcein-AM (33.3 ng/mL; Molecular Probes, Eugene, OR, USA) for 20 minutes at 37°C, washed twice in PBS, and resuspended to a concentration of 1x10^6^/mL in HEPES medium (containing 132 mM of NaCl, 20 mM of HEPES, 6.0 mM of KCl, 1.0 mM of MgSO_4_, 1.0 mM of CaCl_2_, 1.2 mM of potassium phosphate, 5.5 mM of glucose, and 0.5% (wt/vol) human serum albumin, pH 7.4). Labelling of neutrophils did not influence their TEM capacity as compared to unlabeled cells (data not shown).

All experiments involving human blood samples were conducted in accordance to the Declaration of Helsinki. The study was approved by the local ethical committees of the Amsterdam University Medical Center and Sanquin Blood Supply, Amsterdam, The Netherlands.

### SDS-PAGE and Western Blot Analysis

Total cell lysates were prepared from freshly purified neutrophils. Samples were separated by SDS polyacrylamide gel electrophoresis and transferred onto a nitrocellulose membrane. Individual proteins were detected with antibodies against ARPC1B (rabbit polyclonal, Sigma-Aldrich, St Louis, MO, USA) and actin (mouse monoclonal, Sigma-Aldrich).

Secondary antibodies were donkey anti-mouse-IgG IRDye 800CW or donkey-anti–rabbit-IgG IRDye 680LT (LI-COR Biosciences, Lincoln, NB, USA). Visualization of bound antibodies was performed on an Odyssey Infrared Imaging system (LI-COR Biosciences).

### Neutrophil Adhesion (Static Condition)

Neutrophils (5x10^6^/mL) were labeled with calcein-AM (1 µM; Molecular Probes, Eugene, OR, USA) for 30 minutes at 37°C, washed twice in PBS, and resuspended to a concentration of 2x10^6^/mL in HEPES medium. Neutrophil adhesion was determined on an uncoated 96-well MaxiSorp plate (Nunc, Wiesbaden, Germany) in response to numerous stimuli as described previously (4).

### Flow Cytometry

Flow cytometry was performed to assess the expression of various neutrophil surface markers. Fluorescein isothiocyanate (FITC)-, allophycocyanin (APC), phycoerythrin (PE), Brilliant Violet 510 (BV510)-, or Alexa Fluor 647 (AF647)-labelled monoclonal antibodies and isotype controls were used according to the instructions of the manufacturer. Antibodies were CD18-FITC (mouse IgG1 clone MEM48, Diaclone, Besançon cedex, France), CD11a-FITC (mouse IgG2a, Sanquin reagents, Amsterdam, The Netherlands), CD11b-FITC (mouse IgM clone CLB-mon-gran/1, B2, Sanquin reagents), CD11c-FITC (mouse IgG1 clone BU15, Bio-Rad, Kidlington, UK) CD66b-FITC (mouse IgG1 clone CLB-B13.9, Sanquin reagents), L-selectin-APC (mouse IgG clone DREG-56, BD Pharmingen, San Diego, CA, USA), CD177-FITC (mouse IgG1 clone MEM-166, Bio-Rad), FPR1-FITC (mouse IgG2a clone 350418, R&D Systems, Minneapolis, MN, USA), TNFRI-PE (mouse IgG1 clone 16803, R&D Sytems), TNFRII-APC (mouse IgG2A clone 22235, R&D Sytems), TLR4-APC (mouse IgG2A clone HTA125, Invitrogen), ICAM-1-AF647 (mouse IgG1 clone 15.2, Bio-Rad), PECAM-1-BV510 (mouse IgG1 clone WM59, BD Biosciences, San Jose, CA, USA), JAM-A (mouse IgG1 clone WK9, Thermo Fisher Scientific, Waltham, MA, USA).

Samples were analyzed on a FACSCanto-II flow cytometer using FACS-Diva software (BD Biosciences). Neutrophils were gated based on their forward- and side scatter. Per sample, 10,000 gated events were collected. Data were analyzed with FACS-Diva software.

### Endothelial Cell Culture

Pooled human umbilical vein endothelial cells (HUVEC P1052; Lonza, Basel, Switzerland, cat C2519A) were cultured at 37°C with 5% CO_2_ in fibronectin-coated 10 cm tissue culture plastic petri dishes in Endothelial cell growth medium (EGM-2; PromoCell, Heidelberg, Germany, cat C22011) supplemented with supplementMix (Promocell, cat C39216). The HUVECs were passaged at 60-70% confluency and used for experiments at passage 3-4.

### Transendothelial Migration Under Physiological Flow Conditions

Neutrophil transendothelial migration under flow conditions was assessed as described previously (7), with the exception of labeling of endothelial junctional VE-cadherin and PECAM-1. Neutrophils were flowed over the endothelium for a total of 45 minutes. To induce inflammation, HUVECs were stimulated with 10 ng/mL tumor necrosis factor-alpha (TNF-α; Peprotech, London, United Kingdom) overnight for 16 hours. Neutrophil migration was analyzed using IMARIS Bitplane software (Version 9.5/9.6). Tracking was done using assisted automatic tracking of the neutrophils using manual parameters to classify superendothelial and subendothelial neutrophils. Speed is calculated as the scalar equivalent of the object velocity. We used the track speed mean = average velocity of the spots over time according to the IMARIS reference manual 9.2.0. TEM time was analyzed using Fiji (ImageJ, version 1.52).

### Collagen Gel Preparation

All following steps were carried out on ice to halt polymerization of the collagen. 50 µl of 10x PBS (Gibco, Thermo Fisher Scientific, cat 70011-044) was added to of 400 µl of bovine collagen type-1 (10 mg/mL FibriCol; Advanced BioMatrix, San Diego, CA, USA, cat 5133) and gently mixed. Subsequently, pH was set to 7.4 using 48.6 µl of 0.1M NaOH and checked. The collagen was then diluted 1:1 with medium to achieve a final collagen concentration of 4 mg/mL for vessel-on-a chip or 3D collagen experiments.

### Migration in 3D Collagen Matrix

The 8 mg/mL neutralized collagen was mixed 1:1 with HEPES medium containing 8*10^6^ neutrophils and complement component 5a (C5a; 10 nM; Sino Biological, Wayne, PA, USA). An 8 µl drop of this mixture was placed in the middle of a well of an µ-Slide 8 Well (Ibidi, Gräfelfing, Germany, cat 80826) and flattened with a coverslip, creating a ±10 µm 3D matrix. The device was placed at 37°C for 30 minutes to allow collagen polymerization before adding 250 µL HEPES medium supplemented with 10 nM C5a (37°C). Neutrophil migration was then assessed using an LSM980 Airyscan2 (ZEISS, Oberkochen, Germany) using a 10x air objective (ZEISS, 420640-9900-000, Objective Plan-Apochromat 10x/0.45). Every 5 seconds an image was taken for 50 minutes in total. For integrin blocking experiments, neutrophils were pre-incubated with 10 µg/mL anti-CD11b monoclonal antibody (mAb) clone 44a and 10 µg/mL anti-CD18 mAb clone IB4 for 20 minutes. These antibodies were isolated from the supernatant of hybridoma clones obtained from the American Type Culture Collection (Rockville, MD, USA). Neutrophil migration through the collagen matrix was analyzed in IMARIS Bitplane software (Version 9.5/9.6). The tracking was performed automatically using the autoregressive motion algorithm and checked manually for accuracy. Speed is calculated as the scalar equivalent of the object velocity. We used the track speed mean = average velocity of the spots over time according to the IMARIS reference manual 9.2.0.

### Perfusable Vessel-on-a-Chip

The vessels-on-a chip were made using the devices developed by the lab of Beebe, as previously described (8). Minor alterations to the protocol were made. Briefly, the devices were coated with 1% PEI (Polysciences Inc., Warrington, PA, USA, #23966) and incubated for 10 minutes at room temperature (RT). Sequentially chambers were coated with 0.1% glutaraldehyde (Merck, Darmstadt, Germany, #104239), washed 5x with water for injection (WFI; Gibco, #A12873-01) and air-dried. Collagen-1 was prepared according to the protocol above. 10 µl Collagen-1 was added to each chamber and polymerized for 30 minutes at 37°C, 5% CO_2_. PBS-drenched cotton balls were added to the device to control humidity of the device. Rods were removed using tweezers and EGM-2 was added to the lumen. HUVECs were washed twice with PBS, trypsinized for 5 minutes, treated with trypsin neutralizing solution (TNS; Lonza, cat CC-5002) and centrifuged for 5 minutes at 200xg. HUVECs were then stained using CellTracker™ Green CMFDA Dye (1 µM; Molecular Probes, cat C7025) according to manufactures protocol, washed twice with PBS and pelleted. HUVECs were then resuspended to a concentration of 15*10^6^ cells/mL. 5µl of cell suspension was added to each lumen and placed in head-over-head at 37°C, 5% CO_2_ for 2 hours at 1 RPM. Vessels were matured for 2 days with medium replacement twice daily.

### Neutrophil Transendothelial Migration and Migration Through Collagen Matrix

To induce inflammation, the vessels were stimulated overnight with 10 ng/mL TNF-α. Before the experiment, excess medium was removed and 2 µl of HEPES medium containing 8*10^6^ DiD labelled neutrophils were added to each vessel. Vessels were incubated for 2.5 hours, washed twice and fixed for 15 minutes using 4% paraformaldehyde. Devices were then imaged using an LSM980 Airyscan2 (ZEISS) using a 10x air objective (ZEISS, 420640-9900-000, Objective Plan-Apochromat 10x/0.45). Analysis was done using IMARIS Bitplane software (Version 9.5/9.6).

### Statistical Analysis

Experimental data were plotted and analyzed by GraphPad Prism V9.0.0 (GraphPad Software, San Diego, CA, USA). Results are shown as mean ± standard deviation. Normality was tested using the Shapiro-Wilk test. The paired or unpaired Student *t* test was used to test statistical significance (* = p<0.05; ** = p<0.01; *** = p<0.001; ns = non-significant).

## Results

### ARPC1B-Deficient Neutrophils Display Impaired Subendothelial Motility Upon Transendothelial Migration

We investigated the capacity of neutrophils to transmigrate through an endothelial monolayer under physiological flow conditions. HUVECs were grown on fibronectin, and stimulated with tumor necrosis factor-alpha (TNF-α) which leads to the upregulation of cell adhesion molecules such as ICAM-1 and VCAM-1, as well as the production of important chemoattractants for neutrophils like platelet-activating factor and interleukin-8 (9, 10). Inflamed endothelial cells also release and remodel ECM proteins, including different types of laminins, collagen-I and fibronectin (11).

**Figure 1:**
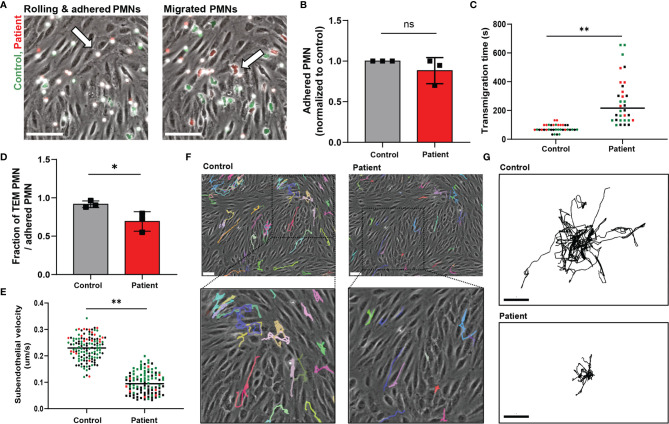
ARPC1B-deficient neutrophils display impaired subendothelial motility upon transendothelial migration. **(A)** Neutrophil TEM through TNF-α inflamed HUVECs was investigated upon physiological flow conditions. Neutrophils (green = control; red = patient) rolled over the endothelium, whereupon they firmly adhered (left panel) and transmigrated (right panel). Neutrophils are circular above the endothelium (left panel, arrow) and become polarized (right panel, arrow) under the endothelium. Representative stills are displayed, see also **Supplementary Video 1**. Scale bar = 100 µm. **(B)** The number of firmly adhered ARPC1B-deficient neutrophils was quantified and normalized to control neutrophils (mean ± SD, n = 3). **(C)** Average time of neutrophils to complete transendothelial migration, starting from firm adherence. Individual cells are depicted. Colors (black, green and red) are corresponding to independent experiments. **(D)** Transendothelial migratory events were quantified and normalized to the number of firmly adhered neutrophils (mean ± SD, n = 3). **(E–G)** Subendothelial motility of transmigrated neutrophils, with **(E)** average velocity of neutrophils (n = 3, individual cells are depicted, colors are corresponding to independent experiments) and cell track analysis of subendothelial neutrophils with **(F)** representative cell trajectories of control and ARPC1B-deficient neutrophils as indicated lasting for 45 minutes (scale bar = 70 µm) and **(G)** showing trajectory plots displayed with their origins brought to a common point. Scale bar = 50 µm. Results are representative of 3 independent experiments. The Student *t* test was used to test statistical significance (*p < 0.05; **p < 0.01; ns, non-significant).

Lack of ARPC1B expression in patient neutrophils was confirmed by Western blotting, which showed the complete absence of ARPC1B protein while actin levels were normal (**Supplementary Figure 1A**). Control and ARPC1B-deficient neutrophils were differently fluorescently labeled and simultaneously perfused over the inflamed endothelium. Control neutrophils rolled over the endothelium, whereupon they firmly adhered and transmigrated (**Figure 1A** and **Supplementary Video 1**). Patient neutrophils rolled and adhered on the endothelium in a similar fashion as control cells (**Figure 1B**), but once arrested, they hardly crawled away from their initial arrest site. Under normal inflammatory conditions, neutrophils crawl on endothelium in order to find suitable sites to transmigrate, mostly at endothelial junctions (12). The observed lack of crawling indicated that patient neutrophils crossed the endothelium at non-optimal locations. Furthermore, ARPC1B-deficient neutrophils remained mostly round-shaped and unable to polarize, which coincided with a significantly decrease in TEM speed as well as a reduced number of neutrophils that successfully crossed the endothelium (**Figures 1C, D**). Both control and ARPC1B-deficient neutrophils transmigrated solely *via* the paracellular pathway (**Supplementary Figure 1B**). The adherence of ARPC1B-deficient neutrophils to the endothelial monolayer under flow conditions is remarkable but well in accordance with our observations that ARPC1B-deficient neutrophils show normal expression of adhesion and signaling receptors, including CD11a (integrin αL chain), CD11b (integrin αM chain), CD18 (integrin β2 chain), L-selectin, PECAM-1 and ICAM-1, as well as adherence under static conditions in response to a range of stimuli (**Supplementary Figure 1C, D**).

Once transmigrated, control neutrophils were actively migrating away from the initial TEM site underneath the endothelium. However, ARPC1B-deficient neutrophils that did cross the endothelium showed a prominent decrease in subendothelial motility (**Figure 1E**). Moreover, they failed to migrate away from their initial TEM site, in contrast to control neutrophils (**Figures 1F, G**). These results indicate that ARPC1B-deficient neutrophils have a minor defect in actual TEM, but a more pronounced defect in their ability to migrate underneath the endothelium.

### Neutrophil Infiltration Into 3D Tissue Matrices Is Defective in ARPC1B Deficiency

**Figure 2:**
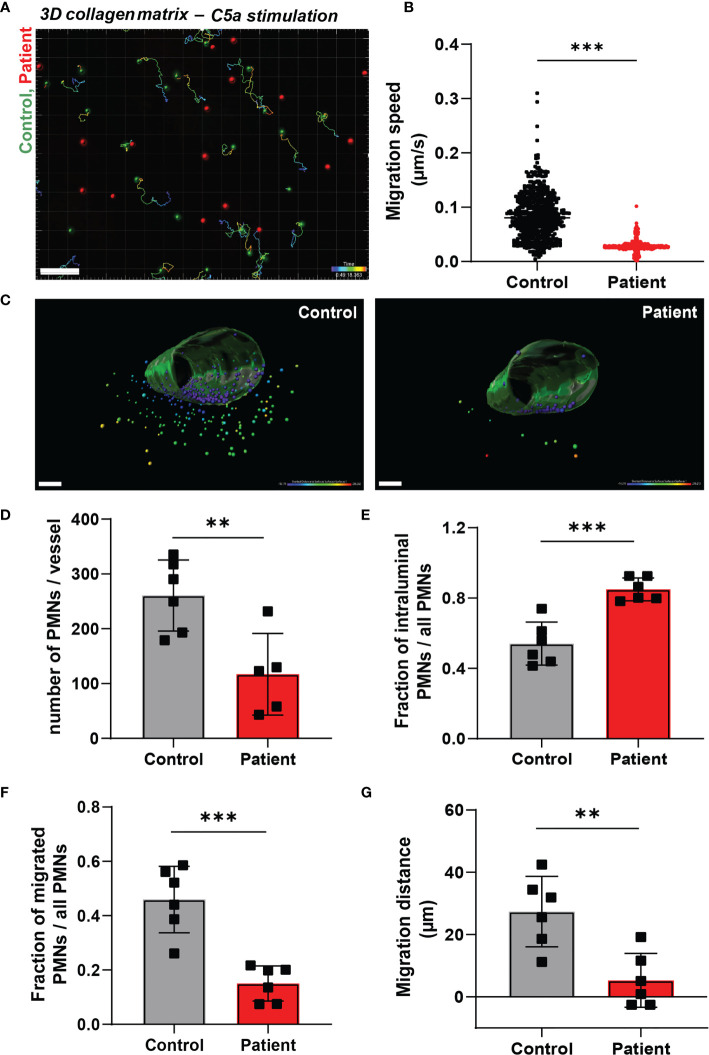
Neutrophil infiltration into 3D tissue matrices is defective in ARPC1B deficiency. **(A)** Motility tracks of neutrophils (green = control; red = patient) in a collagen-I 3D matrix upon C5a stimulation, see also **Supplementary Video 2**. Only control neutrophils show motility tracks as ARPC1B-deficient neutrophils were found to be non-motile. Scale bar = 100 µm. Heat bar = time in minutes. Results are representative of 3 independent experiments. **(B)** Migration speed of neutrophils in collagen matrix (cells of 3 experiments pooled). **(C)** Representative images of neutrophil TEM in a perfusable vessel-on-a-chip, see also **Supplementary Videos 3**, **4**. Scale bar = 100 µm. Heat bar = distance starting from vessel surface. Results are representative of 6 vessels, 2 fields of view per vessel were analyzed. **(D–F)** Quantification of neutrophil TEM using vessel-on-a-chip model, with **(D)** number of neutrophils retrieved in the vessel, **(E)** number of intraluminal neutrophils (normalized to total number of neutrophils), **(F)** number of neutrophils infiltrated into subendothelial collagen matrix (normalized to total number of neutrophils), **(G)** and average migration distance of neutrophils into the vessel. Mean ± SD, results are representative of 6 vessels, 2 fields of view per vessel were analyzed. The Student *t* test was used to test statistical significance (**p < 0.01; ***p < 0.001; ns, non-significant).

The dimensions change for a neutrophil as soon as they enter the area underneath the endothelium, i.e. from a luminal 2D to an ECM 3D setting. It has been previously observed that several types of leukocytes, including granulocytes, are able to efficiently migrate in 3D matrices in an integrin-independent manner (13). We investigated neutrophil motility and migration in an artificial 3D gel of bovine collagen-I and visualized chemokinesis of neutrophils upon activation with the chemoattractant C5a. First, we confirmed the integrin-independency of (control) neutrophils for 3D migration in collagen by usage of blocking monoclonal antibodies directed against the common integrin β2 chain (clone IB4, CD18) or the αM chain (clone 44a, CD11b). Indeed, we did not observe an effect from integrin blockage on 3D migration in collagen I (**Supplementary Figure 2A**), indicating that this mode of migration is independent of the main integrins of neutrophils. Next, we investigated the requirement of ARPC1B in neutrophil migration in the collagen-I 3D gel by visualizing chemokinesis of differently fluorescently labeled control and ARPC1B-deficient neutrophils upon activation with C5a (**Supplementary Video 2** and **Figure 2A**) and TNF-α stimulation (**Supplementary Figure 2B**). Both C5a and TNF-α induced migration of control neutrophils in the collagen matrix. Quantification of migration paths upon C5a stimulation revealed that control neutrophils migrated successfully into the collagen matrix with average speeds of 0.085 um/s up to 0.31 um/s. However, ARPC1B-deficient neutrophils were practically non-motile (**Figure 2B**).

To study both processes, i.e. TEM and 3D matrix migration of neutrophils in one assay, we used a vessel-on-a-chip model (see *Methods*). Neutrophils were injected in the vessel, whereupon they were allowed to adhere and migrate for 2.5 hours before the vessels were washed and fixed. As the vessels were subsequently washed and fixed, non-adherent neutrophils were lost. Of note, no flow was applied on the vessel. Using the 3D vessel-on-a-chip model, we found that the number of ARPC1B-deficient neutrophils retrieved in the vessel was significantly lower than control neutrophils (**Supplementary Videos 3**, **4** and **Figures 2C, D**). Most ARPC1B-deficient neutrophils were found at the intraluminal site firmly adhered to the endothelium, as multiple washing steps did not remove them (**Figure 2E**). Around 45% of the retrieved control neutrophils transmigrated successfully, while only 15% of ARPC1B-deficient neutrophils completed their transendothelial migration route (**Figure 2F**). Neutrophils that did penetrate the matrix were found in closer proximity to the vessel compared to control neutrophils. ARPC1B-deficient neutrophils migrated on average 5 µm into the matrix, while this was almost 30 µm for control neutrophils (**Figure 2G**).

## Discussion

Overall, our results indicate that the ARP2/3 complex is particularly important for motility in a 3D environment subsequent to the endothelial cell layer itself. Yet, the initial steps of rolling and adhesion strengthening under flow conditions seems independent of ARPC1B, allowing transendothelial migration. Of interest, the number of ARPC1B-deficient neutrophils retrieved in the vessel was significantly lower than control neutrophils, though input was equal (**Figure 2D**). This indicates that these patient neutrophils were not firmly adhered to the endothelial cells and lost during washing. This might be explained by the fact that no physiological flow was applied to the vessel-on-a-chip system. Flow forces have been shown to induce forces between adhering cells and substrates that leads to integrin activation and adhesion strengthening (14, 15). This flow-enhanced integrin activity may explain the difference observed between firm adhesion under flow conditions and the static vessel-on-a-chip environment. Alternatively, it might also be that neutrophils which fail to transmigrate over a longer period of incubation in the vessel-on-a-chip approach (2.5 hours) are more prone to detach from the endothelium again compared to the shorter incubations under flow conditions (45 minutes) or static adhesion on plastic (30 minutes). The process of adhesion is energy-demanding and neutrophils which fail to transmigrate might be prone to detach from the endothelium again, perhaps as a result of being deprived from their energy reserves.

Recent attention was raised to the issue of leukocytes employing alternative migration mechanisms depending on 2- or 3 dimensionality (16). The impaired TEM observed when using the vessel-on-a-chip model may thus indicate that ARPC1B is of more importance for migration in 3D environments. Indeed, when neutrophils are flowed over inflamed endothelium, they migrate in a 2D-manner, while under the endothelium they are in a ‘confined’ 3D environment and clearly lack such capacity to move around. This implicates that ARPC1B-deficient neutrophils are able to extravasate, but cannot penetrate through the BM into the tissue. Interestingly, 69% of patients with APRC1B deficiency suffer from cutaneous vasculitis (17). Previous investigations showed that ARPC1B-deficient neutrophils release their azurophilic granules prematurely *in vitro* (4), both of which may contribute to the vascular damage as observed in these patients.

After neutrophils have crossed the endothelium, and before entering the tissue, there is a second layer of cells to cross: the pericytes (18). We were not able to include the role for pericytes in the current study as our vessel-on-a-chip has its limitations and does not allow culturing a second cell layer. However, this would be a future challenge.

Our results emphasize the importance of ARPC1B for neutrophil migration, thereby explaining the severe susceptibly of these rare patients to bacterial infections, while neutrophil killing mechanisms have been found to be intact (4).

## Data Availability Statement

The raw data supporting the conclusions of this article will be made available by the authors, without undue reservation.

## Ethics Statement

The studies involving human participants were reviewed and approved by the local ethical committees of the Amsterdam University Medical Center and Sanquin Blood Supply, Amsterdam, The Netherlands. Written informed consent to participate in this study was provided by the participants’ legal guardian/next of kin.

## Author Contributions

ES wrote the manuscript. all authors designed the experiments. LK, AS, and ES performed experiments. LK and AS analyzed the data. All authors contributed to the article and approved the submitted version.

## Funding

ES and TK are supported by the European Union’s Horizon 2020 research and innovation programme under Grant Agreement No 668303 and TK is supported by the E-Rare ZonMW grant #90030376506. AS is supported by LSBR grant #1649. LK is supported by LSBR grant #1820 JB is supported by ZonMW NWO Vici grant # 91819632.

## Conflict of Interest

The authors declare that the research was conducted in the absence of any commercial or financial relationships that could be construed as a potential conflict of interest.

## Supplementary Material

Supplementary Figure 1
**(A)** Absence of ARPC1B protein and normal actin levels in patient neutrophils was found by Western blot. **(B)** Quantification of neutrophil migration *via* paracellular mode of control- and ARPC1B-deficient neutrophils. **(C)** Expression of adhesion and surface molecules on the neutrophil membrane was assessed by flow cytometry. Neutrophils are gated based on forward/side scatter. Mean fluorescence intensity (MFI) is corrected for the isotype control (mean ± SD, n = 1 – 5). **(D)** Adhesion of neutrophils to plastic (static condition) as percentage of total input upon stimulation with the indicated stimuli (mean + SD, n = 2 - 3).

Supplementary Figure 2
**(A)** Migration speed of neutrophils in collagen matrix in response to C5a with or without blockage of integrin β2 chain (clone IB4, CD18) and the αM chain (clone 44a, CD11b). Individual cells are depicted. Colors (black, green and red) are corresponding to independent experiments. **(B)** Migration speed of control- and ARPC1B-deficient neutrophils in collagen matrix in response to PBS (negative control), C5a or TNF-α. Individual cells are depicted, n = 1.

Supplementary Video 1 Neutrophil TEM through inflamed endothelium. Fluorescently labelled neutrophils (green = control; red = ARPC1B-deficient patient) were flowed over TNF-α inflamed HUVECs for 45 minutes, see also **Figure 1A**. Scale bar = 80 µm. Results are representative of 3 independent experiments.

Supplementary Video 2 Neutrophil migration in a collagen-I 3D matrix. Migration of neutrophils (green = control; red = ARPC1B-deficient patient) in a collagen-I 3D matrix upon C5a stimulation, see also **Figure 2A**. Neutrophil motility was assessed for a total of 50 minutes. Only control neutrophils show motility tracks as ARPC1B-deficient neutrophils were found to be non-motile. Heat bar = time in minutes. Results are representative of 3 independent experiments.

Supplementary Video 3 Control neutrophil TEM in a vessel-on-a-chip. Neutrophils were injected in the vessel, whereupon they were allowed to adhere and migrate for 2.5 hours before the vessels were washed and fixed, see also **Figure 2C**. Scale bar = 150 µm. Results are representative of 6 vessels.

Supplementary Video 4 ARPC1B-deficient neutrophil TEM in a vessel-on-a-chip. Neutrophils were injected in the vessel, whereupon they were allowed to adhere and migrate for 2.5 hours before the vessels were washed and fixed, see also **Figure 2C**. Scale bar = 150 µm. Results are representative of 6 vessels.
