# Supplementary figures and images for: Defective Neutrophil Transendothelial Migration and Lateral Motility in ARPC1B Deficiency Under Flow Conditions
Source: Front Immunol. 2021 May 31;12:678030. doi: 10.3389/fimmu.2021.678030 (PMC8202084; doi:10.3389/fimmu.2021.678030)

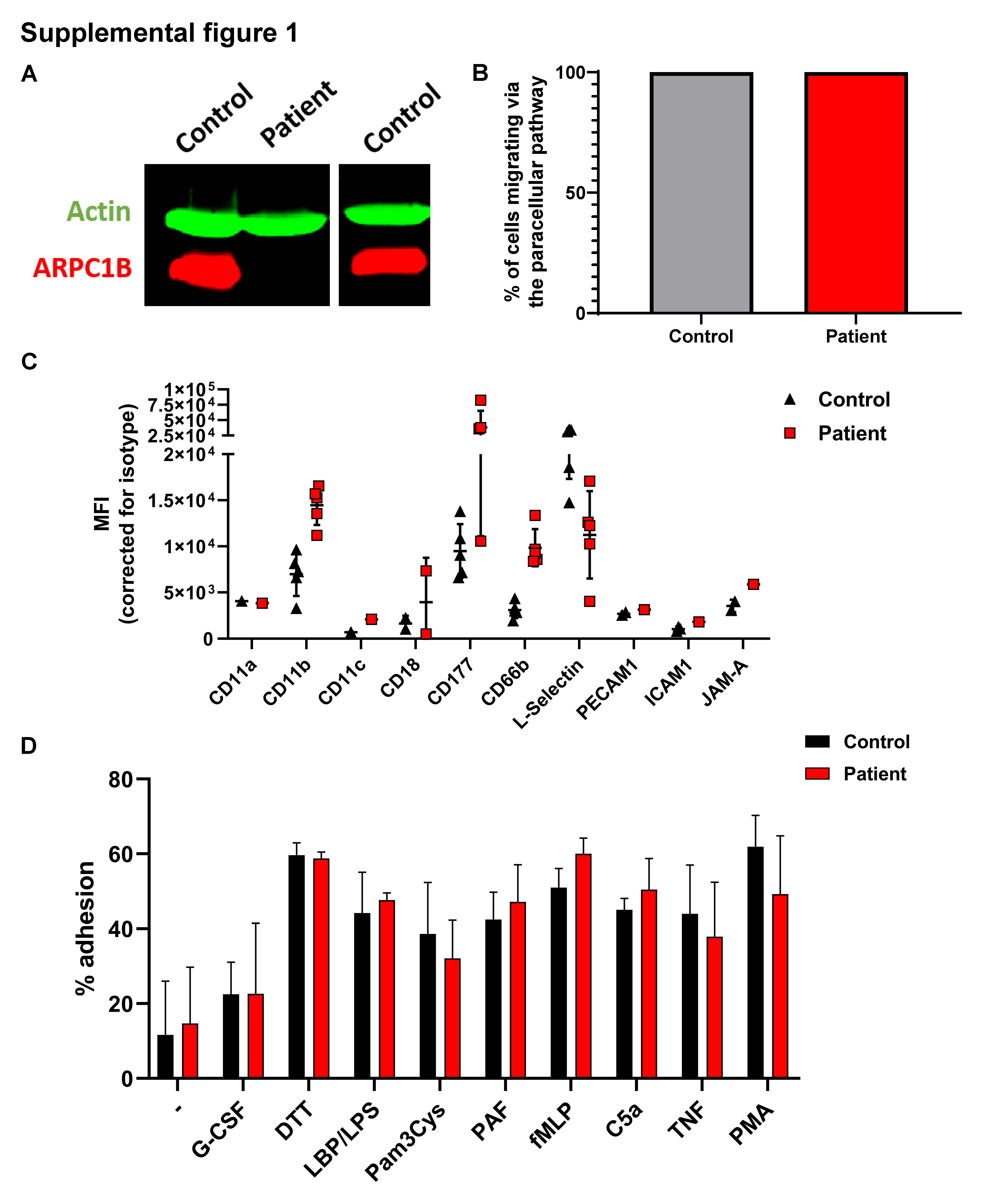

Supplement: Supplementary Figure 1 — (A) Absence of ARPC1B protein and normal actin levels in patient neutrophils was found by Western blot. (B) Quantification of neutrophil migration via paracellular mode of control- and ARPC1B-deficient neutrophils. (C) Expression of adhesion and surface molecules on the neutrophil membrane was assessed by flow cytometry. Neutrophils are gated based on forward/side scatter. Mean fluorescence intensity (MFI) is corrected for the isotype control (mean ± SD, n = 1 – 5). (D) Adhesion of neutrophils to plastic (static condition) as percentage of total input upon stimulation with the indicated stimuli (mean + SD, n = 2 - 3). [file Image_1.tif]

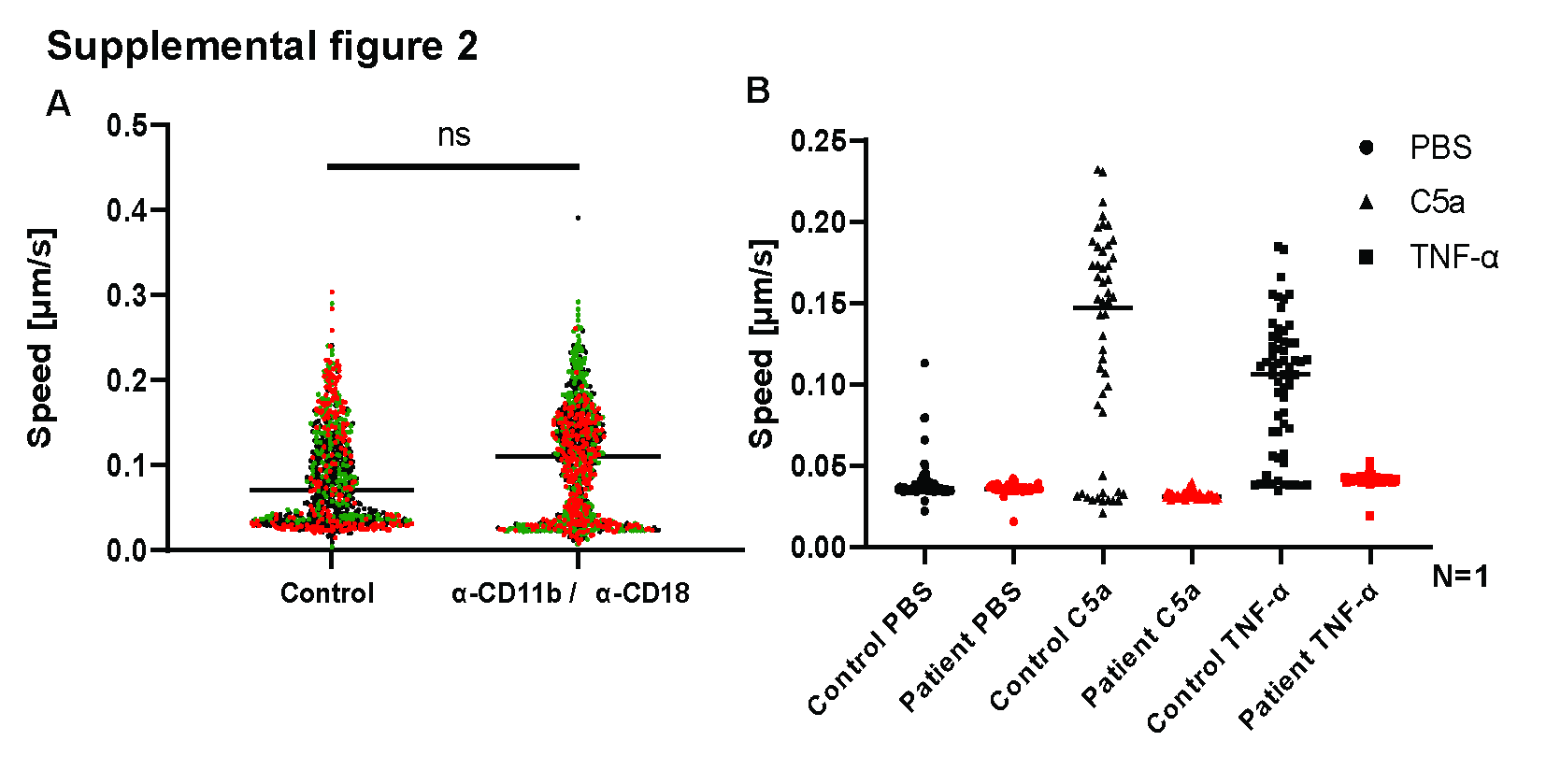

Supplement: Supplementary Figure 2 — (A) Migration speed of neutrophils in collagen matrix in response to C5a with or without blockage of integrin β2 chain (clone IB4, CD18) and the αM chain (clone 44a, CD11b). Individual cells are depicted. Colors (black, green and red) are corresponding to independent experiments. (B) Migration speed of control- and ARPC1B-deficient neutrophils in collagen matrix in response to PBS (negative control), C5a or TNF-α. Individual cells are depicted, n = 1. [file Image_2.tif]
